# Sex differences in cancer-specific survival for locally advanced esophageal cancer after neoadjuvant chemoradiotherapy: A population-based analysis

**DOI:** 10.3389/fsurg.2022.989204

**Published:** 2022-07-29

**Authors:** Jiaqiang Wang, Chengwei Ye, Chaoyang Zhang, Kaiming Wang, Furong Hong, Qingqin Peng, Zilong Chen

**Affiliations:** ^1^ Department of Radiation Oncology, The First Hospital of Quanzhou Affiliated to Fujian Medical University, Quanzhou, China; ^2^ Department of Gastrointestinal Surgery, The First Hospital of Quanzhou Affiliated to Fujian Medical University, Quanzhou, China

**Keywords:** neoadjuvant chemoradiotherapy, cancer-specific survival, SEER database, esophageal cancer, nomogram model, male, sex difference

## Abstract

**Objective:**

Neoadjuvant chemoradiotherapy (nCRT) is the recommended standard treatment for locally advanced esophageal cancer (LA-EC). This study aimed to determine whether sex makes a difference in cancer-specific survival (CSS) and construct a novel nomogram model to predict CSS for LA-EC after nCRT based on the SEER database.

**Methods:**

Patients coded by 04–15 were identified from the SEER database. Patients with systemic treatment and radiotherapy before surgery were defined as nCRT. We further divided this population into a training group and a verification group at a ratio of 7:3. Univariate and multivariate cox analyses were applied to determine the prognostic risk factors based on the training cohort, and then the Nomogram model was established. The area under the curve (AUC) was used to evaluate the predictive ability of the model. We used the calibration curve to evaluate the consistency between the predicted status and actual status and decision curve analysis (DCA) to evaluate the clinical value. We used X-tile software to determine the best cut-off value of nomogram scores and divided the population into low-risk, medium-risk, and high-risk groups, and Kaplan-Meier analysis was applied to compare the CSS.

**Results:**

A total of 2096 LA-EC patients were included for further analysis, with 1,540 in the training cohort and 656 in the validation group. Male (HR: 1.29, 95% CI, 1.04 −1.58), T stage, N stage, and M stage were identified as independent risk factors of CSS based on the training cohort. A Nomogram model was constructed to predict the 3-, 5- and 7-years CSS. ROC curve and AUC confirmed that this nomogram has median discrimination ability. The calibration curve showed good agreement between predicted status and actual status. The DCA curves confirmed the clinical value. Kaplan-Meier analysis indicated that patients in the high-risk subgroup had poorer CSS in both the training cohort and validation cohort (*P* < 0.001).

**Conclusion:**

Male patients had poorer CSS in LA-EC patients after nCRT. A nomogram model composed of sex, T stage, N stage, and M stage was constructed to identify the high-risk population and provide a personalized follow-up plan.

## Introduction

Esophageal cancer is a highly aggressive malignancy, with a 5-year overall survival (OS) of only 10% to 20% in patients with advanced-stage (1). Compared with the surgery alone group, neoadjuvant chemoradiotherapy (nCRT) could significantly improve overall survival (OS) (100.1 months vs. 66.5 months) and disease-free survival (100.1 months vs. 41.7 months) for locally advanced esophageal cancer (LA-EC) (2). The 10-year OS of the CROSS trial indicated that the absolute benefit of nCRT was 13% (38% vs. 25%) (3). Based on current evidence, nCRT is still the first choice of treatment for LA-EC. Sex is reported to be a clinicopathological feature that could affect long-term survival (4, 5). However, at present, whether sex could affect the survival of LA-EC receiving nCRT is still unclear.

The Union for International Cancer Control tumor/node/Metastasis (TNM) staging system is widely used to predict long-term survival and guide adjuvant therapy, but its identification ability is limited. Sometimes, patients diagnosed with EC have different survival, even with the same TNM stage (4, 5). Nomograms are widely used to effectively predict survival in patients with all types of cancer-based on clinicopathological features (6). Nomogram is a new visualization tool that combines risk factors with other predictors to assess the absolute risk of an individual patient and is widely used to help doctors make decisions. The sample size is an important factor in constructing a reliable nomogram model. Surveillance, Epidemiology, and End Results (SEER) database population is a public population, which contains approximately 35% population of Americans, and could provide enough sample size for model development.

This study aimed to determine whether sex makes a difference in cancer-specific survival (CSS) and construct a novel nomogram model to predict CSS for LA-EC receiving nCRT based on the SEER database population, which could help in risk stratification and provide individualized therapy.

## Methods

We downloaded data from SEER * stat software (version 8.3.6). The study included EC patients who underwent nCRT after esophagectomy between 2004 and 2015. Inclusion criteria: (1) primary EC, (2) preoperative nCRT, (3) sufficient clinicopathological features, demographic data, cause of death, and follow-up information. Exclusion criteria: (1) lack of basic clinical information such as age, sex, and marital status; (2) Lack of pathological information, T stage, N stage, pathological type, histological grade, and cause of death.

The demographic characteristics (age, sex, race, insurance status, and marital status), disease characteristics (histology, primary location, tumor size, grade, t, N, M stage), treatment methods (radiotherapy, chemotherapy), survival time and living status of patients were analyzed. We divided patients into three groups according to tumor size (<51, 51–76, and >76 mm). We divided the patients into three groups (>65 years old, 50 −65 years old, <50 years old). The primary site was defined according to the international classification of tumor diseases Code: lower esophagus 1 / 3 (15.5), middle esophagus 1 / 3 (15.4), upper esophagus 1 / 3 (15.3), and others. The histological types included adenocarcinoma, squamous cell carcinoma, and others. Tumor differentiation was divided into four groups: grade I, grade II, grade III, and grade IV. The population included in this study was staged by the 7th edition TNM stage system.

### Statistical analysis

Univariate and multivariate Cox proportional hazards regression analyses were used to identify independent prognostic factors of CSS. Variables with *P* < 0.05 in univariate analysis were included in multivariate Cox regression for further analysis.We used backward likelihood ratio to select variables in the multivariate Cox regression analysis. The Nomogram model was constructed based on the identified independent risk factors. We used the area under the curve (AUC) to evaluate the predictive ability. Calibration curves were drawn for the prediction of 3-,5-, and 7-year CSS, respectively, and decision curve analysis (DCA) curves were drawn to evaluate the clinical value. Based on the nomogram score, patients were divided into low-, medium-, and high-risk groups in X-tile software. The nomogram model was constructed based on the training cohort and evaluated in both the training cohort and validation cohort. We conducted analysis in R software (version 3.6.1). A two-sided *P* value < 0.05 was defined as statistically significant. The primary endpoint of the study was CSS, defined as the time between the date of diagnosis and the date of cancer death or the date of the last follow-up.

## Results

### Baseline characteristics in the training cohort and validation group

**Figure 1:**
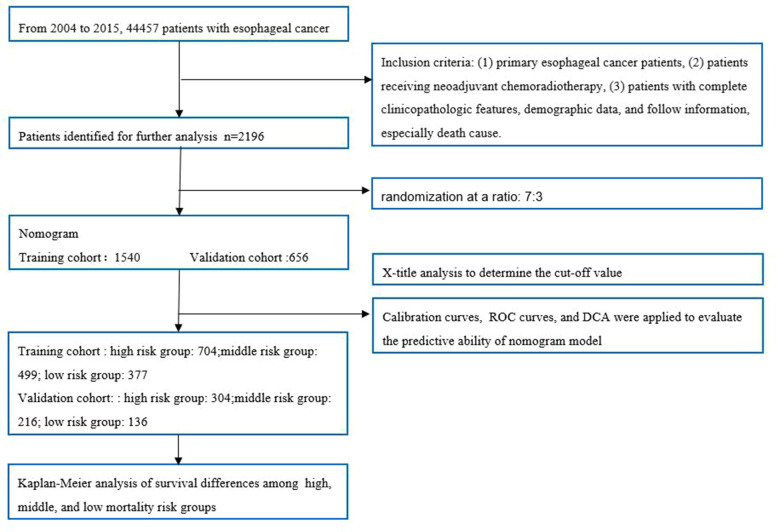
The flow chart of patient selection and data analysis.

A total of 2,096 patients were identified from the SEER database using SEER*Stat Version 8.3.6 software. The details of patient selection were summarized in Figure 1. The total population was divided into a training cohort of 1,540 patients and a validation cohort of 656 patients. The training group and validation group were comparable in baseline characteristics (*P* > 0.05). The comparisons are summarized in Table 1.

**Table 1:** Comparisons of baseline characteristics between training cohort and validation cohort.

Contents	Training cohort	Validation cohort	*P-*value
Number	1540	656	
Race			0.1
Black	75	38	
Other	59	22	
White	1406	596	
Age			0.4
<50	162	61	
50–65	825	371	
>65	553	224	
Sex			0.84
Female	233	97	
Male	1307	559	
Marital status			0.96
Married	1088	464	
Unmarried	452	192	
Tumor size			0.64
< 51 mm	1016	419	
51–76 mm	322	145	
>76 mm	202	92	
T stage			0.5
T1-2	418	169	
T3-4	1122	487	
N stage			0.51
N0	515	229	
N1	1025	427	
M stage			0.66
M0	1402	590	
M1	138	66	
Grade			0.44
Grade I	75	33	
Grade II	675	275	
Grade III	765	341	
Grade IV	21	7	
Histology			0.59
Adenocarcinoma	1110	454	
Squamous cell carcinoma	261	130	
Others	169	72	
Primary site			0.75
Upper	16	6	
Middle	156	72	
Lower	1246	518	
Other	122	60	
Radiotherapy after surgery			0.64
With	58	22	
Without	1482	634	
Chemotherapy after surgery			0.8
With	139	57	
Without	1401	599	

### Development and validation of nomogram model

We used the training cohort to find prognostic risk factors. Univariate analysis indicated that tumor size, M stage, N stage, T stage, grade, and sex were prognostic factors. Multivariate COX analysis determined that M stage (HR = 1.41, 95% CI, 1.14–1.75, *P* = 0.002), N stage (HR = 1.62, 95% CI, 1.39–1.89, *P* < 0.001), T stage (HR = 1.25, 1.06–1.46, *P* = 0.01), and sex (HR = 1.29, 95% CI, 1.04–1.58, *P* = 0.02) were independent prognostic factors. The details of univariate and multivariate Cox analysis were summarized in Table 2.

**Table 2:** Univariate and multivariate Cox analysis of cancer-specific survival for esophageal cancer patients receiving neoadjuvant chemoradiotherapy in the training cohort.

Characteristics	Univariate Cox analysis		Multivariate Cox analysis	
	HR 95% CI	*P*	HR 95% CI	*P*
Age				
<50	reference			
50–65	1.08 (0.87–1.35)	0.48		
>65	1.09 (0.86–1.37)	0.48		
Sex				
Female	reference			
Male	1.36 (1.12–1.67)	0.002	1.29 (1.04–1.58)	0.02
Race				
Black	reference	1.00		
Other	0.86 (0.53–1.37)	0.52		
White	0.99 (0.73–1.34)	0.94		
Martial				
Married	reference			
Marital_Unmarried	1.04 (0.9–1.21)	0.59		
Grade				
Grade I				
Grade II	1.11 (0.79–1.55)	0.56	1.09 (0.77–1.53)	0.63
Grade III	1.45 (1.04–2.02)	0.03	1.37 (0.98–1.92)	0.06
Grade IV	1.94 (1.03–3.66)	0.04	1.69 (0.9–3.18)	0.11
Histology				
Adenocarcinoma	reference			
Squamous cell carcinoma	0.78 (0.65–0.95)	0.01	0.91 (0.75–1.11)	0.36
Other	1.06 (0.86–1.31)	0.58	0.99 (0.8–1.22)	0.91
M stage				
M0	reference			
M1	1.44 (1.16–1.78)	0.001	1.41 (1.14–1.75)	0.002
N stage				
N0	reference			
N1	1.71 (1.47–1.99)	<0.001	1.62 (1.39–1.89)	<0.001
Primary site				
Upper	reference			
Middle	1.46 (0.64–3.34)	0.37		
Lower	1.58 (0.71–3.52)	0.27		
Other	2.1 (0.91–4.81)	0.08		
T stage				
T 1-2	reference			
T 3-4	1.38 (1.18–1.61)	<0.01	1.25 (1.06–1.46)	0.01
Tumor size				
<51 mm	reference			
51–76 mm	1.24 (1.05–1.46)	0.01	1.1 (0.93–1.3)	0.27
>76 mm	1.3 (1.07–1.58)	0.008	1.21 (1–1.48)	0.05
Radiotherapy after surgery				
without	reference			
with	1.15 (0.82–1.61)	0.41		
Chemotherapy after surgery				
without	reference			
with	0.83 (0.67–1.03)	0.09		

**Figure 2:**
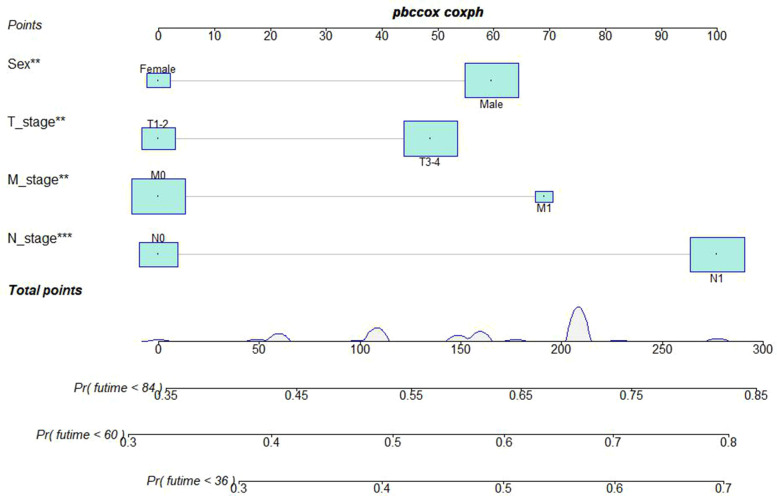
Nomogram model to predict the cancer-specific survival (CSS) at 3-,5-, and 7- years in patients receiving neoadjuvant chemoradiotherapy.

**Figure 3:**
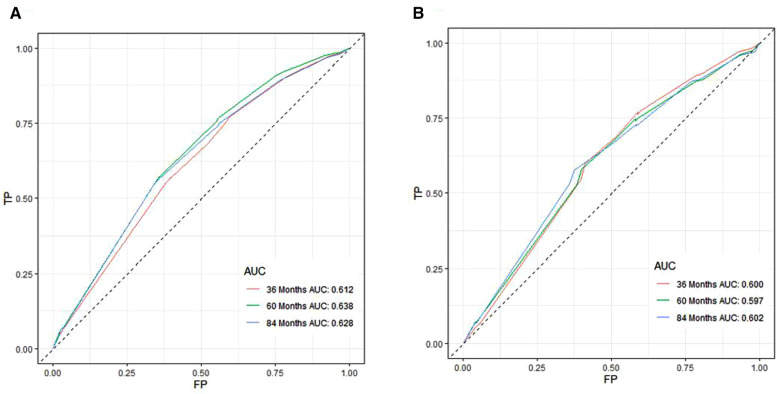
ROC curves for CSS prediction of patients receiving neoadjuvant chmoradiotherapy. (**A**) ROC curves of 3-, 5-, and 7-years in the training cohort, (**B**) ROC curves of 3-, 5-, and 7-years in the validation cohort. TP, True positive rate; FP, false positive rate; ROC, Receiver operating characteristic; CSS, cancer-specific survival.

**Figure 4:**
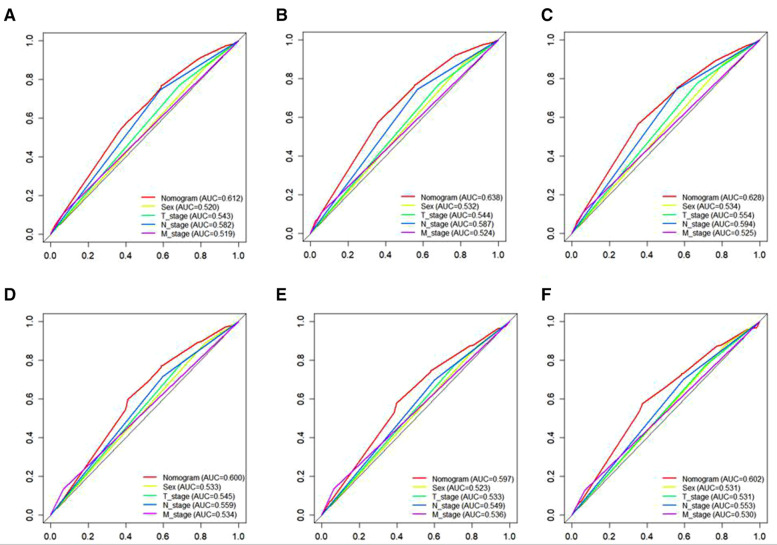
The ROC curves for CSS, including the nomogram model and all independent predictors at 3- (**A**), 5- (**B**), and 7-years (**C**) in the training cohort and at 3- (**D**), 5- (**E**), and 7-years (**F**) in the validation cohort. ROC, Receiver operating characteristic; CSS, cancer specific survival.

**Figure 5:**
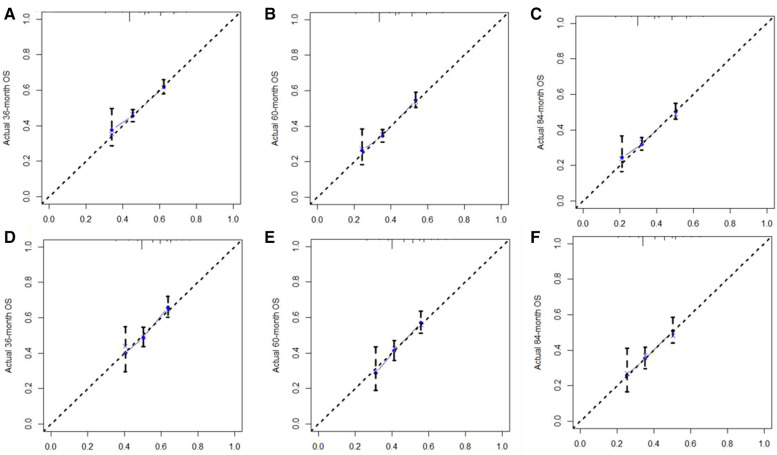
The calibration curve for predicting CSS at (**A**) 3-years, (**B**) 5-years, (**C**) 7-years in the training cohort, and at (**D**) 3-years, (**E**) 5-years, (**F**) 7-years in validation cohort. The nomogram-predicted probability of CSS is plotted on the X-axis, and the actual CSS is plotted on the Y-axis. CSS: cancer-specific survival.

**Figure 6:**
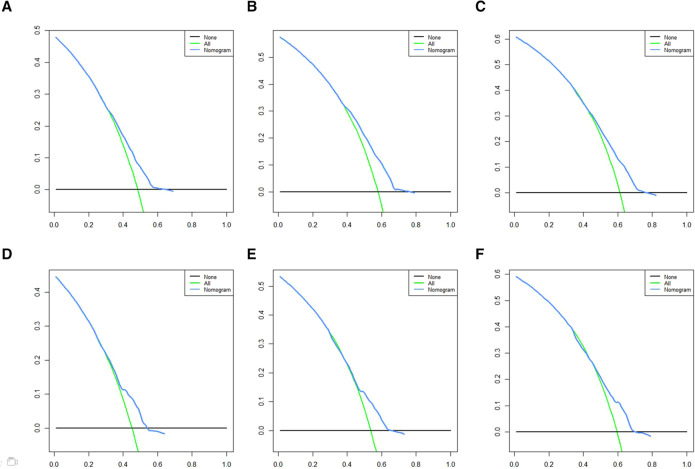
DCA for CSS prediction. (**A**) DCA of 3-years CSS in the training cohort, (**B**) DCA of 5-years CSS in the training cohort, (**C**) DCA of 7-years CSS in the training cohort, (**D**) DCA of 3-years CSS in the validation cohort, (**E**) DCA of 5-years CSS in the validation cohort, (**F**) DCA of 7-years CSS in the validation cohort. DCA, Decision curve analysis; CSS, cancer-specific survival.

A Nomogram model was developed to predict 3-, 5-, and 7-years CSS (Figure 2). The AUC for 3-,5-, and 7-years CSS was 0.612,0.638, and 0.628 respectively in the training cohort, and 0.597,0.60, and 0.602 respectively in the validation cohort. Time-dependent ROCs noted that this model performed well in predicting CSS in both the training cohort and validation cohort (Figure 3) and also had a higher prediction accuracy than individual prognostic factors included in the model (Figure 4). The calibration curves indicated that the predicted survival status was highly consistent with the actual status in both training and validation cohort (Figure 5). DCA indicated that this nomogram model had strong clinical applicability (Figure 6).

### Kaplan-Meier analysis and risk stratification

**Figure 7:**
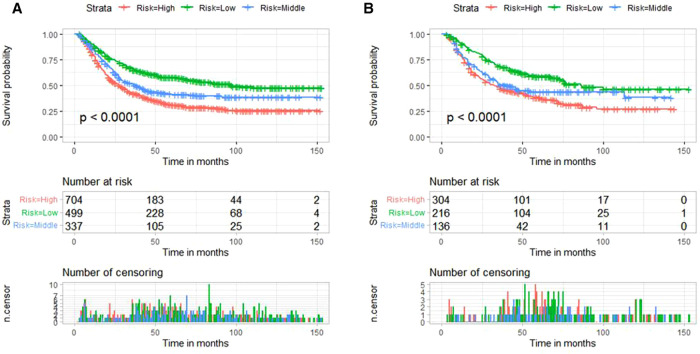
Risk stratification based on nomogram score and Kaplan-Meier curves for cancer-specific survival in training cohort (**A**) and validation cohort (**B**).

Using X-tile software, patients were divided into low-risk, medium-risk subgroups, and high-risk subgroups according to nomogram scores. Nomogram scores of 0–5 are defined as a low-risk group, 6–10 as a medium-risk group, and 11–14 as a high-risk group. Compared with the low-risk group, the relative risk of the medium-risk group and high-risk group were 1.28 and 1.51, respectively. In the validation cohort and training cohort, patients in the low-risk group had significantly better CSS (*P* < 0.001) **(**Figure 7**).**

## Discussion

To our knowledge, there is significant heterogeneity in the individual survival rate of EC, and the prediction of cancer-specific survival rate using the AJCC staging system alone seems to be inaccurate and inadequate. Although the AJCC staging system is the most widely used system for prognostic assessment and clinical treatment of cancer patients (7). However, due to a lack of demographic information, the AJCC system is not a perfect predictor of CSS in EC patients. Previous studies have confirmed that age at diagnosis, gender, race, marital status, and occupation are significantly associated with cancer survival (8–10). In the establishment of prognostic models for patients with EC, the prognostic value is limited due to the relatively limited sample size (11, 12). We found that sex also played an important role in CSS of EC patients receiving nCRT, and we further developed a richer and more accurate prognostic model (including T stage, N stage, M stage, and sex) to predict CSS. The nomogram could be used to calculate individual CSS predictions and provide better treatment allocation. Based on the nomogram, we could divide patients into low-, medium-, and high-risk, and a personalized follow-up plan could be conducted.

Male was an independent risk factor for poor CSS in EC receiving nCRT. Whether there is a sex difference in survival is still conflicting. Nobel TB et al. reported that postoperative mortality and overall survival (OS) were similar between sexes. In patients with clinical stage II/III, females received neoadjuvant therapy less frequently than males and had worse survival (13). Recently, Ji Zhang et al. found that women had a lower excess mortality rate ratio of 0.76 in EAC subtypes and 0.52 in ESCC based on 1,301 patients from Sweden nationwide. In patients with neoadjuvant therapy, the sex difference benefits still persisted (14). Kauppila JH et al. found that the women had better long-term survival than men in the ESCC subtype but not in the EAC subtype (15). Rowse PG et al. found that after induction chemoradiotherapy, the male sex had an 80% increased risk of recurrence (hazard ratio 1.80, *P* = 0.008) (16). Estrogen receptors (ERs) are highly expressed in ESCC, and estrogens were reported to inhibit squamous cell tumor growth (17, 18). However, age-stratified studies did not show better survival in younger women with higher sex hormone levels. Other key prognostic factors for EC involve alcohol consumption, smoking consumption, obesity, lifestyle, and oncogenic types of HPV (19). The female sex could respond better to induction chemoradiotherapy. The response difference may be due to sex-related differences in pharmacokinetics and pharmacodynamics (20). The mechanism is still unclear and should be further explored.

Based on the 7th AJCC staging system, differentiation grade is a staging factor for EC. He W et al. also reported that although patients with poorly differentiated EC respond better to nCRT than those with well-differentiated or moderately differentiated EC, however, resulted in poorer survival (21). For EC patients with the same pathological stage, a worse pathological grade often indicates a worse prognosis and a higher postoperative recurrence rate (22). However, we found that pathological grade wasn't an independent risk factor of CSS for EC receiving nCRT, which was consistent with the 8th AJCC staging system. One possible reason is that the cell redistribution or loss of original morphology after neoadjuvant therapy affects the judgment of pathological grade, which would reduce the value of pathological grade in predicting survival.

At present, the number of population-based EC patients after NCRT is still relatively limited. This study clarified the value of gender differences in CSS for EC patients after nCRT and established a new model to predict the CSS of EC patients after nCRT at 3, 5, and 7 years. However, this study had the following three limitations: first, this study is based on the SEER database. Due to the differences in demographic characteristics and pathological subtypes, it was uncertain whether the conclusions of this study are applicable to the Asian population. Secondly, there was no record of a chemoradiotherapy regimen in the SEER database. The radiotherapy or chemotherapy dose described in SEER data was yes or no/unknown. We defined a combination of preoperative systemic therapy and preoperative radiotherapy as nCRT. There weren’t surgical method, R0 removal rate, and number of lymph nodes removed in the SEER database. Third, this novel model was only verified internally, not externally. The findings of this study should be further verified in later research.

## Conclusions

Male patients had poorer CSS in LA-EC patients after nCRT. A nomogram model composed of sex, T stage, N stage, and M stage was constructed to identify the high-risk population and provide a personalized follow-up plan.

## Data Availability

The original contributions presented in the study are included in the article/Supplementary Material, further inquiries can be directed to the corresponding author/s.
